# Actin nucleator formins regulate the tension-buffering function of caveolin-1

**DOI:** 10.1093/jmcb/mjab070

**Published:** 2021-10-27

**Authors:** Xuemeng Shi, Daijiao Tang, Yifan Xing, Shuangshuang Zhao, Changyuan Fan, Jin Zhong, Yanqin Cui, Kun Shi, Yaming Jiu

**Affiliations:** The Joint Program in Infection and Immunity, Guangzhou Women and Children’s Medical Center, Guangzhou Medical University, Guangzhou 510623, China; The Joint Program in Infection and Immunity, Institut Pasteur of Shanghai, Chinese Academy of Sciences, Shanghai 200031, China; Unit of Cell Biology and Imaging Study of Pathogen Host Interaction, The Center for Microbes, Development and Health, CAS Key Laboratory of Molecular Virology and Immunology, Institut Pasteur of Shanghai, Chinese Academy of Sciences, Shanghai 200031, China; College of Life Sciences, University of Chinese Academy of Sciences, Beijing 100049, China; College of Life Sciences, University of Chinese Academy of Sciences, Beijing 100049, China; Unit of Viral Hepatitis, CAS Key Laboratory of Molecular Virology, Institut Pasteur of Shanghai, Chinese Academy of Sciences, Shanghai 200031, China; The Joint Program in Infection and Immunity, Guangzhou Women and Children’s Medical Center, Guangzhou Medical University, Guangzhou 510623, China; The Joint Program in Infection and Immunity, Institut Pasteur of Shanghai, Chinese Academy of Sciences, Shanghai 200031, China; Unit of Cell Biology and Imaging Study of Pathogen Host Interaction, The Center for Microbes, Development and Health, CAS Key Laboratory of Molecular Virology and Immunology, Institut Pasteur of Shanghai, Chinese Academy of Sciences, Shanghai 200031, China; College of Life Sciences, University of Chinese Academy of Sciences, Beijing 100049, China; College of Life Sciences, University of Chinese Academy of Sciences, Beijing 100049, China; Unit of Viral Hepatitis, CAS Key Laboratory of Molecular Virology, Institut Pasteur of Shanghai, Chinese Academy of Sciences, Shanghai 200031, China; The Joint Program in Infection and Immunity, Guangzhou Women and Children’s Medical Center, Guangzhou Medical University, Guangzhou 510623, China; The Joint Program in Infection and Immunity, Institut Pasteur of Shanghai, Chinese Academy of Sciences, Shanghai 200031, China; The Joint Program in Infection and Immunity, Guangzhou Women and Children’s Medical Center, Guangzhou Medical University, Guangzhou 510623, China; The Joint Program in Infection and Immunity, Institut Pasteur of Shanghai, Chinese Academy of Sciences, Shanghai 200031, China; The Joint Program in Infection and Immunity, Guangzhou Women and Children’s Medical Center, Guangzhou Medical University, Guangzhou 510623, China; The Joint Program in Infection and Immunity, Institut Pasteur of Shanghai, Chinese Academy of Sciences, Shanghai 200031, China; Unit of Cell Biology and Imaging Study of Pathogen Host Interaction, The Center for Microbes, Development and Health, CAS Key Laboratory of Molecular Virology and Immunology, Institut Pasteur of Shanghai, Chinese Academy of Sciences, Shanghai 200031, China; College of Life Sciences, University of Chinese Academy of Sciences, Beijing 100049, China

**Keywords:** caveolin-1, elongated actin filaments, formins, hypo-osmotic shock, matrix stiffness

## Abstract

Both the mechanosensitive actin cytoskeleton and caveolae contribute to active processes such as cell migration, morphogenesis, and vesicular trafficking. Although distinct actin components are well studied, how they contribute to cytoplasmic caveolae, especially in the context of mechano-stress, has remained elusive. Here, we identify two actin-associated mobility stereotypes of caveolin-1 (CAV-1)-marked intracellular vesicles, which are characterized as ‘dwelling’ and ‘go and dwelling’. In order to exploit the reason for their distinct dynamics, elongated actin-associated formin functions are perturbed. We find drastically decreased density, increased clustering, and compromised motility of cytoplasmic CAV-1 vesicles resulting from lacking actin nucleator formins by both chemical treatment and RNA silencing of formin genes. Furthermore, hypo-osmosis-stimulated diminishing of CAV-1 is dramatically intensified upon blocking formins. The clustering of CAV-1 vesicles when cells are cultured on soft substrate is also aggravated under formin inhibition condition. Together, we reveal that actin-associated formins are essential for maintaining the dynamic organization of cytoplasmic CAV-1 and importantly its sensitivity upon mechanical challenge. We conclude that tension-controlled actin formins act as a safety valve dampening excessive tension on CAV-1 and safeguarding CAV-1 against mechanical damage.

## Introduction

All living cells are constantly exposed and respond to a myriad of dynamic mechanical cues, such as contraction, stretch, osmosis shock, and extracellular stiffness change, that play a central role in the control of various cellular functions including proliferation, differentiation, migration, and apoptosis (Kumar et al., 2017). Caveolae are flask-shaped pits on plasma membrane that are mechanosensitive and abundant in many mammalian cell types (Parton, 2018; Parton et al., 2018). Caveolae are mainly composed of caveolin and cavin proteins. Caveolins are the most essential proteins in caveolae to form the integrative structure (Parton and del Pozo, 2013). There are three isoforms of caveolins, caveolin-1 (CAV-1), caveolin-2 (CAV-2), and caveolin-3 (CAV-3), which show dramatic expression differences from tissue to tissue. Caveolae/CAV-1 are involved in a wide range of cellular physiological processes, including endocytosis (Doherty and McMahon, 2009), lipid storage and homeostasis (Parton and Simons, 2007; Martin, 2013), and cell signaling transduction (Parton and Simons, 2007).

In addition to these well-known functions, intensive investigations have targeted the role of caveolae/CAV-1 in mechanosensing and mechanoprotection (Parton and del Pozo, 2013; Del Pozo et al., 2021). The ability to sense membrane forces enables caveolae/CAV-1 to function as a vital plasma membrane sensor (Del Pozo et al., 2021). When cells are facing osmotic swelling or uniaxial stretching, a rapid flattening will occur in caveolar invagination, mainly by caveolae disassembly and release of CAV-1 (Sinha et al., 2011). Increased Tyr14 phosphorylation of CAV-1 and membrane lipid alterations were also detected in this hypo-osmotic process (Sinha et al., 2011). Cells are unable to buffer membrane tension changes in the absence of caveolae/CAV-1, causing membrane rupture and finally cell death (Cheng et al., 2015). When substrate stiffness changes, the activity of the small GTPase Rho, which regulates actin cytoskeleton assembly, is necessary, but not sufficient, for CAV-1-dependent mechanoregulation of the yes-associated protein (YAP) activity (Moreno-Vicente et al., 2018). These findings raise the possibility that caveolae/CAV-1 alteration might be a general mechanism for cells to respond to various mechanical stimuli where cytoskeleton network has been tightly associated to. However, the identification and functional analysis of specific cytoskeleton components, which are involved in regulating the process of caveolae/CAV-1-mediated tension-sensing, have not been carefully carried out.

As one of the most important cell components, actin cytoskeleton is highly associated with many cellular physiological processes, including membrane ruffles, filopodia formation, and endocytosis (Le et al., 2020). Linearized actin assembly, stress fiber, can sense and transmit mechanical inputs via cell–extracellular matrix (ECM) and cell–cell adhesions due to its high sensitivity to mechanical forces (Romet-Lemonne and Jégou, 2013; Wu et al., 2017; Puleo et al., 2019). When cells are under osmotic stress, focal adhesion kinase (FAK) can be phosphorylated, indicating that actin–focal adhesions (FAs) machinery (Wickström et al., 2010) are involved in the osmo-stasis regulation (Lunn and Rozengurt, 2004). Previous studies revealed the intimate links between caveolae/CAV-1 and stress fibers (Echarri and Del Pozo, 2015). Our previous work also found that CAV-1 knockout led to disordered stress fibers and prominent lamellipodia by regulating the active level of RhoA–myosin II and Rac1–PAK1–Cofilin (Shi et al., 2021). Inhibition of stress fibers, but not Arp2/3-dependent branched actin filaments, diminished the phosphorylation of CAV-1 on site Tyr14 (Shi et al., 2021). It has been reported that CAV-1-regulated actin-related mechanosensitive pathways was closely related to its regulation on RhoA activity (Peng et al., 2007). CAV1 has also been shown to regulate integrin-mediated actin remodeling of FA formation, mainly through Rac1 and Cdc42 (Grande-Garcia et al., 2007). These processes are all associated with cell mechanosensing and mechanotransduction, making us think about the specific mechanism involved.

Formins are large multidomain actin-binding proteins that mediate linearized filament nucleation and rapid elongation of long-straight actin filaments. Assembly of many cellular structures, such as filopodia and stress fibers, depends on the diverse and specialized activities of formins (Courtemanche, 2018; Le et al., 2020). Importantly, formins were also shown to possess the ability of mechanosensing (Zimmermann and Kovar, 2019). There is a proposed model that formin–actin complex might represent an elementary mechanosensing device responding to force by enhancement of actin assembly (Kozlov and Bershadsky, 2004). In agreement with this, the actin-nucleating formin factor mDia1 was shown to stabilize the actin cytoskeleton and resist tension through mDia1-dependent actomyosin contractility (Acharya et al., 2017; Fessenden et al., 2018), suggesting that actin-nucleating factors can provide a target for cells to regulate the actin cytoskeleton upon mechanical stress. Moreover, by using viscous drugs and the microfluidics, it was found that the elongation rate of surface-anchored formin mDia1 is increased up to two folds by the application of a pulling force (Higashida et al., 2013; Jégou et al., 2013). During filopodia adhesions, formin proteins are required, most probably through a role in the transmission of force through the actin core (Alieva et al., 2019). However, the relationship between formins and CAV-1 in the context of tensional environment has not been explored.

In this study, we identified that the dynamics of cytoplasmic CAV-1 were tightly associated with actin filaments, which were categorized into two groups termed as ‘dwelling’ and ‘go and dwelling’ according to their movement patterns. Formin inhibitor and siRNA treatment targeting pronounced formin factors FHOD1 and Dia1 resulted in enlarged CAV-1 vesicles, reduced number of CAV-1 vesicles, and slow movement speed. When cells were challenged by hypo-osmotic shock or grown on softer matrix, the number of CAV-1 vesicles gradually dropped to resist the stress. Importantly, compromised formin by either its inhibitor or knockdown of FHOD1 and Dia1 expedited the disappearance of CAV-1 significantly in both processes. Together, our study identified the ‘sensitivity controller’ role of formin to CAV-1, in the context of challenges of osmotic shock and substrate stiffness pressure.

## Results

### There is a tight spatial–tempol association between elongated actin filaments and CAV-1

**Figure 1:**
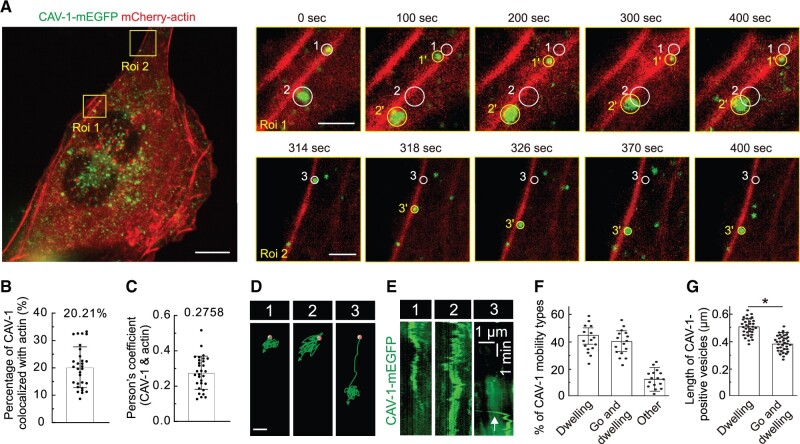
The association of actin filaments with cytoplasmic CAV-1. (**A**) Time-lapse imaging of U2OS cells expressing CAV-1-mEGFP and mCherry-actin revealing the motility of cytoplasmic CAV-1 vesicles along actin filaments. Two magnified regions (yellow boxes-defined Roi1 and Roi2) are chosen. White and yellow circles in the magnified regions indicate the starting and ending positions of discrete CAV-1-tagged vesicles. Scale bar, 10 µm (in cell image), 2 µm (magnified yellow box Roi 1 image), and 2 µm (magnified yellow box Roi 2 image), respectively. (**B**) The percentage of CAV-1 colocalized with actin. *n* = 30 cells are used for quantification. (**C**) Pearson’s coefficient of CAV-1 and actin. *n* = 30 cells are used for quantification. (**D**) Moving trajectories of CAV-1-positive vesicles 1, 2, and 3 in A. Red dots and green arrows indicate the starting and ending points. Scale bar, 1 μm. (**E**) The kymograph analysis of CAV-1-positive vesicles 1, 2, and 3 in **A**. White arrow in vesicle 3 illustrates the fast-moving trail of CAV-1 signals. The vertical scale bar represents 1 min and the horizontal scale bar represents 1 µm. (**F**) The percentage of distinct mobility types of cytoplasmic CAV-1. *n* = 16 cells are used for quantification. The data are presented as mean ± SEM. (**G**) The mean length of ‘dwelling’ (*n* = 34 vesicles) and ‘go and dwelling’ (*n* = 37 vesicles) CAV-1-positive vesicles. Data are represented as mean ± SEM. *P ≤ 0.05 (*t*-test).

To study the dynamic regulation of CAV-1 caveolae, human osteosarcoma cells were used, which display an extensive actin network and easily recognizable CAV-1 vesicles, enabling determination of the extent of correlation between these structures (Figure 1A). Although there is a certain amount (not substantially low) of CAV-1 colocalized with actin filaments in U2OS cells (Figure 1B and C), live cell imaging enabled us to discriminate between two different behavior patterns of the CAV-1 vesicles, one where CAV-1 vesicles were relatively immotile showing only negligible movement and the other one where they rapidly moved for longer distances along filamentous actin before stopping. We coined the first category ‘dwelling’ and the second ‘go and dwelling’. In Figure 1A, the dwelling-type of CAV-1-mEGFP is shown, representing both individual caveolae pits and rosettes (circles 1 and 2 in Roi 1), and the ‘go and dwelling’ category is illustrated in Roi 2, where movement of a fluorescent CAV1-dot (circle 3 in Roi 2) was observed for 12 sec while traveling ∼4 μm along filamentous actin before it suddenly stopped and remained stationary for the rest of the video sequence (Figure 1A, D, and E; Supplementary Video S1).

For subsequent quantification, similar observations of CAV-1 in a number of live cells by videoing at a frequency of 1 frame/sec for 10 min period enabled us to define the ‘dwelling’ category to represent fluorescent spots that did not undergo displacements larger than 0.2 μm during 300 sec of observation, whereas the ‘go and dwelling’ category required a minimal velocity of 0.2 μm/sec in the ‘go’-phase with a subsequent phase of ‘dwelling’ for 50–200 sec. The situations where the CAV-1-mEGFP fluorescence did not apply to these definitions were classified as ‘other’. Using the above definitions and documenting 324 CAV-1 vesicles associated with actin filament bundles in 16 U2OS-cells, nearly equal proportions of ‘dwelling’ and ‘go and dwelling’ vesicles were revealed while 12% of CAV-1 vesicles were classified as ‘other’ (Figure 1F). We then determined the average size of the two categories of CAV-1 vesicles and found that ‘dwelling’ vesicles displayed a slightly larger length than the ‘go and dwelling’ (Figure 1G), indicating a possible correlation between motility and size of cytoplasmic CAV-1 vesicles. Together, these results suggest that the linearized actin filaments are closely associated with CAV-1 vesicles.

### Actin nucleator formins regulate the organization and dynamics of CAV-1 vesicles

**Figure 2:**
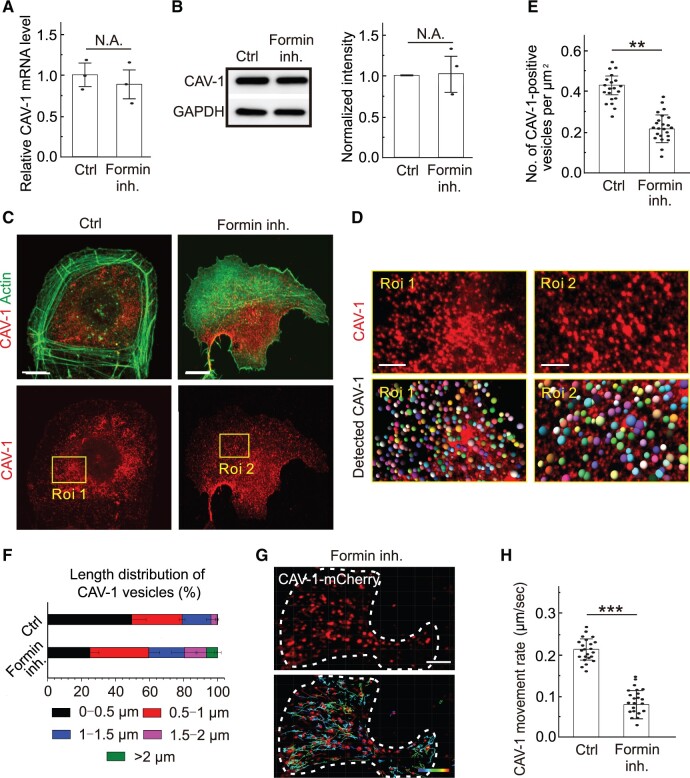
Inhibition of formin results in reduced density and motility of cytoplasmic CAV-1. (**A** and **B**) The transcriptional level of CAV-1 (**A**) and the translational level of CAV-1 and cavin-1 (**B**) are examined by real-time quantitative PCR and western blotting analysis in the wild-type U2OS (Ctrl) cells and formin-inhibited cells. GAPDH is probed for equal sample loading. (**C**) Immunofluorescence staining of endogenous F-actin and CAV-1-positive vesicles in wild-type and formin-inhibited cells. Scale bar, 10 µm. (**D**) Magnified images indicated by yellow box in **C** illustrate the changes of CAV-1 vesicles. Scale bar, 4 µm. The representative analysis of CAV-1-positive dots was detected by Imaris. Identified dots are marked as balls, which are randomly colored in the lower panel. The size of the color balls indicates the calculated CAV-1 vesicle size. (**E**) Quantification of the number of CAV-1-positive vesicles per µm^2^ in the Ctrl (*n* = 20) and formin inhibition (*n* = 24) groups. The data are presented as mean ± SEM. **P ≤ 0.01 (*t*-test). (**F**) The length distribution of CAV-1-positive vesicles. The number of vesicles in each group of size is divided by the total CAV-1 number of the same cell. *n* = 25602 vesicles from 32 cells (Ctrl) and 12035 vesicles from 30 cells (formin inhibition). Data are represented as mean ± SEM. (**G**) The representative dot tracking analysis of CAV-1-positive vesicles in formin-inhibited cells by Imaris. White dashed line indicates the outline of the cell. Color-coded bar from blue to red indicates the tracked mean speeds ranging from 0 to 0.3 μm/sec. Scale bar, 10 μm. (**H**) Quantification of the movement rate of CAV-1-marked vesicles in Ctrl (*n* = 23) and formin-inhibited (*n* = 22) cells. The data are presented as mean ± SEM. ***P ≤ 0.001 (*t*-test).

Having established that actin filaments is tightly associated with the movement of CAV-1 vesicles prompted us to test the influence of these elongated actin assembly arrangements on CAV-1 vesicle behavior. Bundles of linear actin filaments are assembled by formin-driven elongation (del Pozo et al., 2005; Chhabra and Higgs, 2007; Kozera et al., 2009; Gervasio et al., 2011; Sinha et al., 2011; Jiu et al., 2019). We hence used the pharmacological inhibitor SMIFH2 to target formins and thereby interfere with the formation of linear filament assemblies. To avoid disruption of stress fibers and related confounding effects, SMIFH2 was not used at a routine concentration of 25–100 µM but at 15 µM to inhibit formin-induced elongation without completely disrupting the actin stress fibers (Girao et al., 2008; Nassoy and Lamaze, 2012). This drug did not affect the transcription and expression of CAV-1 (Figure 2A and B), while fluorescence imaging witnessed their expected effects on actin organization with extensive loss of filament bundles after exposure to SMIFH2 (Figure 2C). It is also interesting to note that in the cell leading area, where there were normally less actin bundles but more branched actin filaments, formed lamellipodia, and endocytosis happening, there were less CAV-1-tagged vesicles (Figure 2C).

Fluorescence microscopy revealed that formin inhibition caused a significant reduction of CAV-1 vesicles, and the reduced number of vesicles was accompanied by size enlargement (Figure 2D–F). Moreover, live cell imaging demonstrated that the movement of cytoplasmic CAV-1 vesicles was significantly compromised after formin inhibition (Figure 2G and H). Together, these results demonstrate that formin-dependent actin filament assembly is required for the intracellular CAV-1 behavior.

### Depletion of formin proteins FHOD1 and Dia1 compromises the organization and dynamics of CAV-1 vesicles

**Figure 3:**
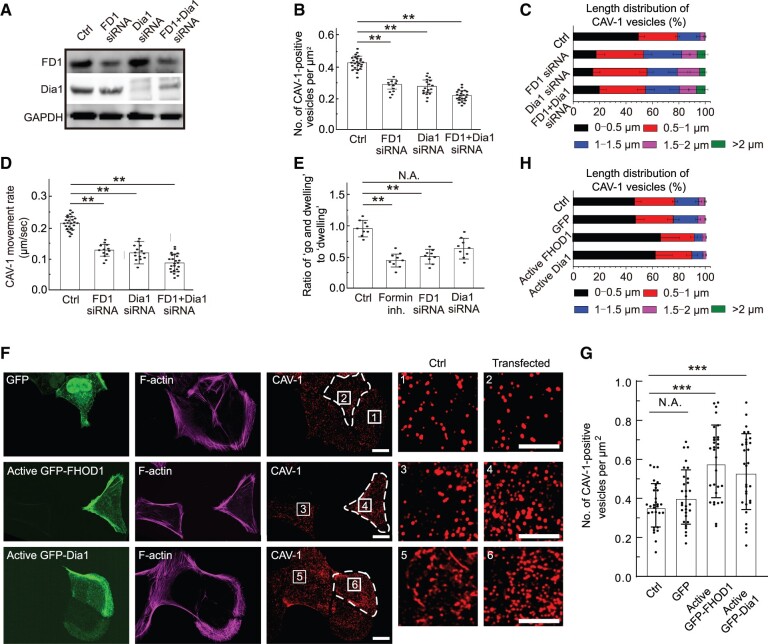
The effects of formin components FHOD1 and Dia1 on the density, size, and motility of cytoplasmic CAV-1 vesicles. (**A**) Western blotting analysis of endogenous FHOD1 and Dia1 levels in total cell lysates of wild-type (Ctrl), FHOD1 knockdown (FD1 siRNA), Dia1 knockdown (Dia1 siRNA), and FHOD1/Dia1 double knockdown (FD1 + Dia1 siRNA) U2OS cells, respectively. GAPDH is probed for equal sample loading. (**B**) Quantification of the number of CAV-1-positive vesicles per µm^2^ in the Ctrl (*n* = 24), FD1 siRNA (*n* = 12), Dia1 siRNA (*n* = 18), and FD1 + Dia1 siRNA (*n* = 23) U2OS cells. The data are presented as mean ± SEM. **P ≤ 0.01 (*t*-test). (**C**) The length distribution of CAV-1-positive vesicles. The number of vesicles in each size group is divided by the total CAV-1 number of the same cell. *n* = 17602 vesicles from 24 cells (Ctrl), 6432 vesicles from 12 cells (FD1 siRNA), 9543 vesicles from 18 cells (Dia1 siRNA), and 10231 vesicles from 23 cells (FD1 + Dia1 siRNA). Data are represented as mean ± SEM. (**D**) Quantification of the movement rate of CAV-1-marked vesicles in the Ctrl (*n* = 25), FD1 siRNA (*n* = 13), Dia1 siRNA (*n* = 14), and FD1 + Dia1 siRNA (*n* = 24) cells. (**E**) Quantification of the ratio of ‘go and dwelling’ to ‘dwelling’ in the Ctrl (*n* = 9), formin-inhibited (*n* = 9), FD1 siRNA (*n* = 9), and Dia1 siRNA (*n* = 9) cells. The data are presented as mean ± SEM. **P ≤ 0.01 (*t*-test). (**F**) Immunofluorescence staining of endogenous F-actin and CAV-1-positive vesicles in U2OS cells expressing GFP, active GFP-FHOD1, and active GFP-Dia1, respectively. Scale bar, 10 and 5 µm in images and magnified images, respectively. (**G**) Quantification of the number of CAV-1-positive vesicles per µm^2^ in each group. *n* = 30 cells. The data are presented as mean ± SEM. ***P ≤ 0.001 (*t*-test). (**H**) The length distribution of CAV-1-positive vesicles. The number of vesicles in each size group is divided by the total CAV-1 number of the same cell. *n* = 16597 vesicles from 20 cells (Ctrl), 15737 vesicles from 20 cells (GFP), 20623 vesicles from 20 cells (active GFP-FHOD1), and 19405 vesicles from 20 cells (active GFP-mDia1). Data are represented as mean ± SEM.

Since the specificity of the inhibitor SMIFH2 has been questioned (Nishimura et al., 2021), to confirm the drug application results, we knocked down FHOD1 and Dia1 (Figure 3A), two major formin variants for actin bundle organization in U2OS cells (Shi et al., 2019). We further used synthetic siRNAs to reduce their expression simultaneously and observed in all cases similar defects of cytoplasmic CAV-1 distribution and dynamics as after formin inhibitor treatment (Figure 3A–D).

Since ‘dwelling’ vesicles show negligible movement for relatively long-time scale, it is logically that the alteration of average movement rates largely depended on the ‘go and dwelling’ vesicles. We thus calculated the ratio of ‘dwelling’ to ‘go and dwelling’ CAV-1 tagged vesicles in U2OS cells treated with formins inhibitor. The quantification revealed that inhibition of formins reduced the proportion of ‘go and dwelling’ CAV-1 tagged vesicles (Figure 3E). The hypothesized inversely correlation between size and motility of CAV-1 vesicles was well evidenced in drug treatment condition. Furthermore, the changes of size and number of CAV-1 vesicles can be reproduced when inhibiting formins in HeLa cells (data not shown).

In addition, we overexpressed the active form of FHOD1(1–1011) and Dia1(ΔN3; 543–1192) fused with GFP, respectively (Watanabe et al., 1999; Shi et al., 2019), and found that the density of CAV-1 in active GFP-FHOD1 and GFP-Dia1 increased by ∼1.62-fold and ∼1.48-fold, respectively, compared to wild-type cells (Figure 3F and G). Furthermore, smaller CAV-1-positive vesicles were found in formin-overexpressing cells (Figure 3H). Taken together, these results indicate that formins, which are critical for the linear elongated actin filaments, play key roles in regulating the CAV-1-tagged vesicles.

### Formins are critical for the tension-sensing function of CAV-1 when cells are challenged by hypo-osmotic shock

**Figure 4:**
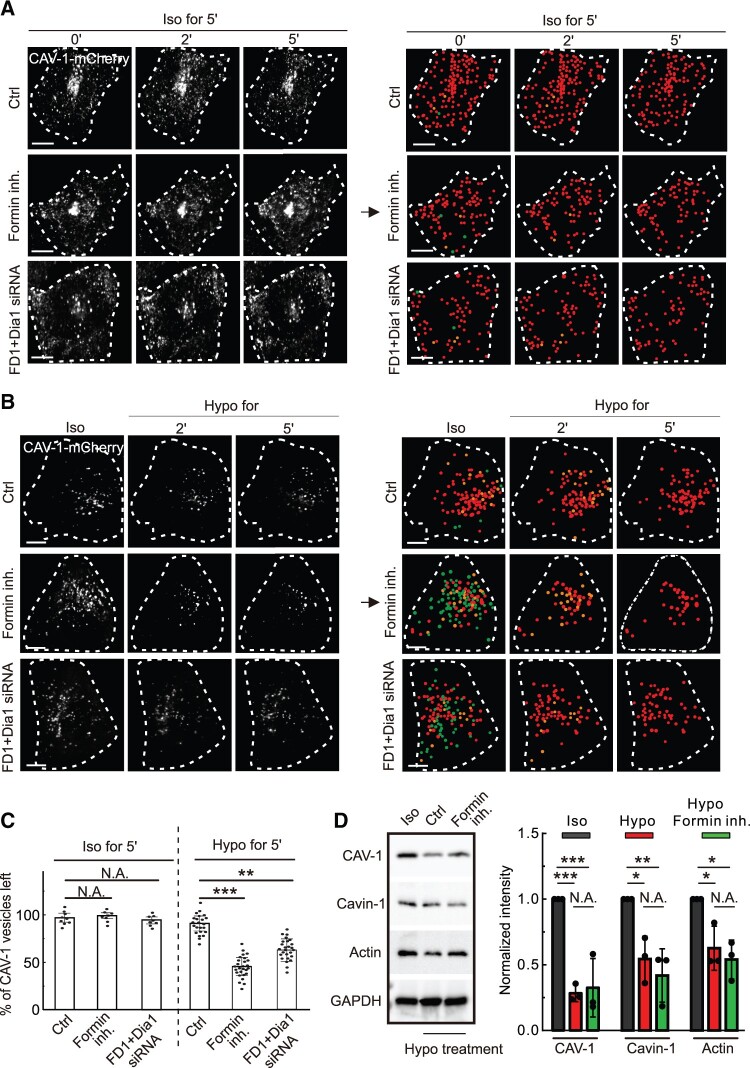
The linear elongated actin network by formins is critical for CAV-1 disappearance upon hypo-osmotic shock. (**A**) Time-lapse imaging of U2OS cells expressing CAV-1-mCherry cultured under routine culture condition (iso-osmosis) with formin inhibition or FHOD1 + Dia1 double knockdown, respectively. (**B**) Time-lapse imaging of U2OS cells expressing CAV-1-mCherry upon hypo-osmotic shock under Ctrl, formin inhibition, or FHOD1 + Dia1 double knockdown condition, respectively. In **A** and **B**, white dash lines indicate the outline of the cells. CAV-1-positive vesicles were detected by Imaris at different time points. Vanished CAV-1 vesicles upon 2- and 5-min iso-osmotic (**A**) or hypo-osmotic (**B**) shock are labeled by green and orange dots, respectively. Red dots illustrate the remaining CAV-1-positive vesicles after 5-min hypo-osmotic treatment. Scale bar, 10 µm. (**C**) Quantification of the percentage of CAV-1-positive vesicles left upon 5-min hypo- or iso-osmotic shock under Ctrl, formin inhibition, or FHOD1 + Dia1 double knockdown conditions, respectively. The data are presented as mean ± SEM. **P ≤ 0.01, ***P ≤ 0.001 (*t*-test). (**D**) Western blotting analysis of CAV-1, cavin-1, and actin levels in U2OS cells with formin inhibition upon hypo-osmotic shock. The blot is also probed with GAPDH antibody to verify equal sample loading. The obtained intensity value from wild-type cells was set to 1. *n* = 3. *P < 0.05, **P < 0.01, ***P < 0.001 (one-way ANOVA).

Caveolae/CAV-1 are reported to function as the mechanosensor, buffering the plasma membrane under tension due to hypo-osmotic stress by causing CAV-1 vesicles to be flatten and fuse with the membrane and therefore seemingly disappear (Sinha et al., 2011). Live cell imaging analysis verified that the number of CAV-1 vesicles gradually dropped by 10% after 5 min upon hypo-osmotic stress (Figure 4A and B).

Our above results indicated that loss of formin activities caused delocalization and altered movement of cytoplasmic CAV-1 vesicles (Figures  2C–H and 3A–D). This prompted us to investigate whether formin-dependent actin filament elongation is involved in the disappearance of CAV-1 vesicles when cells were exposed to osmotic shock. For this, cells were exposed to hypo-osmotic conditions after treatment with SMIFH2. We noted reduced protein levels of CAV-1, cavin-1, and actin upon hypo-osmotic shock but the inhibition of formin did not further affect the amount of these proteins (Figure 4D). By live cell imaging, we witnessed that formin inhibition, either by drug or siRNA, expedited the disappearance significantly by ∼50% after 5 min under hypo-osmotic culture condition (Figure 4B and C). In contrast, under iso-osmotic culture condition, the number of CAV-1 vesicles was not apparently reduced (Figure 4A), excluding the possibility that CAV-1-positive signals were photobleached during live cell imaging. Together, these results suggest that loss of the linear elongated actin filaments regulated by formins makes CAV-1 vesicles more sensitive upon the hypo-osmotic shock.

### Formins are critical for the tension-sensing function of CAV-1 when cells grow on softer matrix

**Figure 5:**
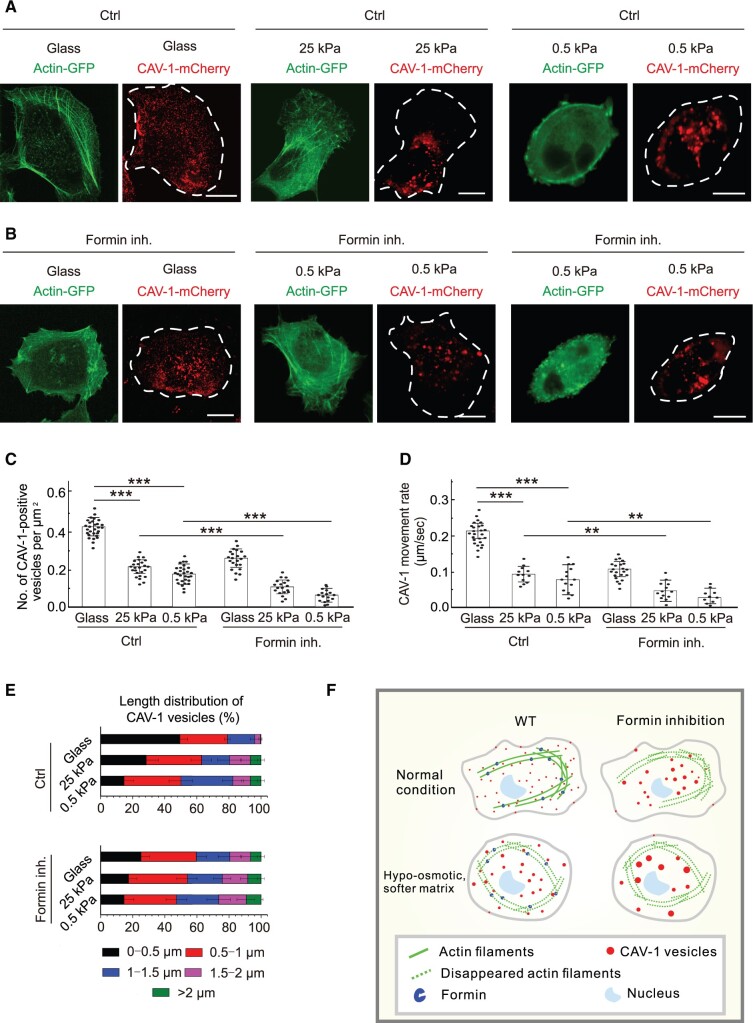
Inhibition of formin promotes the reduced density, increased size, and decreased motility of cytoplasmic CAV-1 vesicles when grown on soft matrix. (**A** and **B**) Representative live cell imaging of actin and CAV-1-positive vesicles in U2OS cells grown on glass or soft substrate 25 or 0.5 kPa, with or without formin inhibitor treatment, respectively. Scale bar, 10 µm. (**C**) Quantification of the number of CAV-1-positive vesicles per µm^2^ in the cells grown on glass (*n* = 29 for Ctrl, *n* = 22 for formin inhibition) or 25 kPa (*n* = 25 for Ctrl, *n* = 19 for formin inhibition) and 0.5 kPa (*n* = 30 for Ctrl, *n* = 17 for formin inhibition) substrates. The data are presented as mean ± SEM. ***P ≤ 0.001 (*t*-test). (**D**) The length distribution of CAV-1-positive vesicles. The number of vesicles in each size group is divided by the total CAV-1 number of the same cell. *n* = 25602 vesicles from 32 Ctrl cells and 16786 vesicles from 29 formin-inhibited cells (glass), 13467 vesicles from 31 Ctrl cells and 8546 vesicles from 33 formin-inhibited cells (25 kPa), and 14567 vesicles from 32 Ctrl cells and 7658 vesicles from 34 formin-inhibited cells (0.5 kPa). Data are represented as mean ±SEM. **P < 0.01, ***P ≤ 0.001 (*t*-test). (**E**) Quantification of the movement rate of CAV-1-positive vesicles in the cells grown on glass (*n* = 26 for Ctrl, *n* = 22 for formin inhibition) and 25 kPa (*n* = 12 for Ctrl, *n* = 13 for formin inhibition) and 0.5 kPa (*n* = 14 for Ctrl, *n* = 10 for formin inhibition) substrates. The data are presented as mean ± SEM. (**F**) The schematic working model. CAV-1 vesicles are associated with and can move on actin filaments in normal cells. Formin inhibition results in decreased CAV-1 vesicle numbers, enlarged vesicle areas, and slowed movement speeds. When cells are challenged with hypo-osmotic shock or softer matrix, CAV-1 vesicles become less in number, larger in area, and slower in speed in order to resist the external environment pressure. Formin inhibition expedites the changes of CAV-1 vesicles in all aspects.

It is known that caveolae rosettes increase upon loss of cell adhesion, which is also accompanied by a profound change in actin organization (Echarri et al., 2012). We observed that cells grown on matrices (25 and 0.5 kPa) softer than glass showed disorganized and fragmented actin bundles compared to cells cultured on glass (Figure 5A). Analogously to the observations above during impaired actin filaments, the CAV-1 vesicles had a bigger size and lower density and displayed slower motility in cells cultured on softer substrates (Figure 5B), supporting our view that caveolae clustering and immobilization resulted from disabling linearized elongation of actin filaments. Cells became rounder and no obvious stress fibers were observed on 0.5 kPa matrix compared with 25 kPa matrix. Meanwhile, the size of the CAV-1 vesicles got bigger (Figures  2C and 5A), and formin inhibitor increased the enlargement of CAV-1 rosettes (Figure 5A and B). Together, these results suggest that loss of actin-associated formin in soft matrix makes CAV-1 vesicles more sensitive upon ECM change.

## Discussion

In this study, we demonstrate the following findings: (i) there is a tight spatial–tempol association between actin network and cytoplasmic CAV-1; (ii) the movement pattern of actin-associated CAV-1 can be divided into two categories: ‘dwelling’ and ‘go and dwelling’; (iii) actin nucleator formins regulate the organization and dynamics of CAV-1 vesicles, loss of which results in enlarged but reduced number of CAV-1 vesicles and smaller movement velocity; (iv) when cells are challenged by hypo-osmotic shock and grown on softer substrate, dropped CAV-1 vesicles number, enlarged CAV-1 vesicles area, and slowed movement dynamics can be observed; (v) formin inhibition expedites the CAV-1 changes in the context of hypo-osmotic shock and substrate stiffness pressure. Taken together, we identify and decipher the role of formins in the regulation of CAV-1 vesicles upon distinct mechano-stimulation mainly by microscopic experiments.

Both previous work and our results here showed that silencing stress fiber regulators mDia1 formin and Abl kinases could induce caveolae clustering and bigger size (Echarri et al., 2012). In addition, mDia1 together with other cytoskeletal proteins regulates the transfer of CAV-1 from microtubules to the cortical actin (Wickström et al., 2010; Echarri et al., 2012). CAV-1α also is shown to control cilium length by modulating Rho GTPase activity via ROCK and Dia1 (Rangel et al., 2019). Above findings suggest a functional coupling of formins with CAV-1 dynamics.

Regarding the relation between formins and CAV-1 in tension-buffering function, a study indicated that CAV-1 depletion decreases the intraepithelial motility, and this effect functions through FMNL2, as the effects of CAV-1 depletion are ameliorated by depleting FMNL2 (Katsuno-Kambe et al., 2021). Similarly, another research revealed that CAV-1 knockdown enhances junctional tension through the junctional F-actin cytoskeleton (Teo et al., 2020). They found that junctional formin FMNL2 was increased in CAV-1 knockdown cells, while the whole protein level of FMNL2 remained unchanged. Moreover, shFMNL2 in CAV-1 knockdown cells specifically restored junctional actin dynamics to the normal levels, suggesting that formin FMNL2 is critical for increased tension caused by CAV-1 knockdown (Teo et al., 2020). Using traction force microscopy, it was found that the strain energy and the maximum exerted traction stress were reduced after shSMIFH2 treatment (Eftekharjoo et al., 2019). Although many studies showed that both CAV-1 and formins could control the cell tension-buffering function, no work has reported the regulation of CAV-1 by formins in the context of mechanosensing and mechanoprotection before. Our work points out the ‘sensitivity controller’ function of formin to CAV-1, giving an explanation of how cells respond to external environmental pressures.

In addition to regulating the movement of CAV-1 vesicles, previous studies mainly focused on formins’ ability to mediate endosomes mobility in an actin filaments-dependent manner. Some Rho GTPases were hypothesized to interact with formin proteins to mediate actin polymerization. During this process, endosomal dynamics can also be affected (Ridley, 2006). For example, hDia2C, a member of the formin-related family, is a RhoD effector. hDia2C and RhoD could regulate endosome–cytoskeleton interactions and reduce endosome motility, mainly requiring Src-kinase activity (Gasman et al., 2003). Another study revealed that RhoB GTPase interacts with its effector protein, formin mDia2, on epidermal growth factor (EGF)-containing endosomes. Using EGFP-tagged Dia-autoregulatory domain to activate endogenous mDia proteins significantly impair the movement of Texas Red-EGF-labeled vesicles. The mechanism is that formin-mediated actin assembly would exert force on vesicles from multiple directions and thus interfere short-range endosome movement (Wallar et al., 2007). Subsequent study showed that the movement of exocrine secretory vesicles to the apical membrane needs actin cables, and the interaction between RhoA and mDia1 may be necessary in the actin cable growth (Geron et al., 2013). FMNL2 and FMNL3 knockout caused a significant increase in average endosome size and abnormal lysosome morphology. Moreover, FMNL2/3 depletion also affected anterograde transport of VSV-glycoprotein from the Golgi to the plasma membrane downstream of Cdc42 (Kage et al., 2017). This finding links formins with trans-medial Golgi or endo- and lysosomes, widening our understanding on formin function. Recent studies revealed that vesicle trafficking in primary cilia and polarity formation in pollen needs formin DIAPH1 and AtFH5, respectively (Palander and Trimble, 2020; Liu et al., 2021). In general, our work expands the understanding of formin function in regulating CAV-1 vesicle movement.

Besides formins, there are several factors identified to regulate the mechanosensing ability of caveolae. ATPase Eps15 homology domain-containing 2 was shown to play a pivotal role in the cell adaptation to mechanical perturbations by connecting caveolae mechanosensing with the regulation of gene transcription (Torrino et al., 2018). So far it is unclear whether these regulatory mechanisms are related to formins or not. Another study revealed that caveolae formation requires a formin-binding protein FBP17, a membrane curvature regulator, loss of which results in lack of plasma membrane tension-buffering capacity under osmotic shock, and this process is controlled through c-Abl kinase (Echarri et al., 2019). This work prompted us to think about what is the specific mechanism for formins to control CAV-1 under tension stress, and whether FBP17 or other formin-binding proteins play a role in this process or not.

Despite accumulated information regarding the interplay between actin filaments and caveolae, the elaborate regulatory mechanism on how caveolae/CAV-1 vesicles are linked to actin filaments has not been completely understood. For the time being, only formins and CAV-1 are linked to the cell tension-buffering process. Whether other critical actin-related components, such as Arp2/3 and tropomyosins, are involved in CAV-1-related regulation demands further study. In addition, only osmosis and matrix stiffness as mechano-stimulators were tested in our study. Whether formins and CAV-1 are involved in other mechanosensing processes is unknown.

Together, this is the first time reporting that formins could regulate CAV-1 in the mechanosensing process, mainly by stabilizing and protecting the mechanosensing ability of CAV-1 in the context of tension stresses. Formin inhibition makes CAV-1 change more significantly under pressure challenges. Our work here has enriched the knowledge of how cells responding to stress challenges, providing new evidence for the connection between various cell components, and has certain guiding significance for the research on the mechanical protection of cells in the future.

## Materials and methods

### Cell culture and transfections

Human osteosarcoma (U2OS) cells were maintained in high-glucose (4.5 g/L) Dulbecco’s modified Eagle’s medium (DMEM) (BE12-614F, Lonza) supplemented with 10% fetal bovine serum (FBS; 10500-064, Gibco), 10 U/ml penicillin, 10 µg/ml streptomycin, and 20 mM L-glutamine (from 100× concentration, Gibco) (later referred as complete DMEM) at 37°C in humidified atmosphere with 5% CO_2_. Transient transfections were performed with Fugene HD (Promega) according to manufacturer’s instructions using a Fugene: DNA ratio of 3.5:1. Incubation of 48 h before fixation with 4% paraformaldehyde (PFA) (Wickström et al., 2010) in phosphate-buffered saline (PBS) was used for myosin-18B rescue experiments. For live cell imaging, cells were detached at 24 h posttransfection with 0.25% (*w*/*v*) trypsin–EDTA and plated onto glass-bottomed 35-mm dishes (MatTek Corporation) coated with 10 µg/ml fibronectin (Roche Molecular Biochemicals) diluted in PBS. siRNA experiments were performed with Lipofectamine RNAiMAX (Invitrogen) using 40 nM on-target plus human siRNA of FHOD1 (target sequence 5ʹ-GCGCUUGAGUAUCGGACUU-3ʹ), Dia1 (target sequence 5ʹ-AAGGAGAGCUCUAAGUCUGCC-3ʹ) (Dharmacon), or 40 nM AllStars negative control siRNA (Qiagen).

### Immunofluorescence microscopy

Briefly, cells were fixed with 4% PFA in PBS for 15 min at room temperature (RT), washed three times with 0.2% bovine serum albumin (BSA) in Dulbecco’s PBS, and permeabilized with 0.1% Triton X-100 in PBS for 5 min. Cells were blocked in 1× Dulbecco’s PBS supplemented with 0.2% BSA. Both primary and secondary antibodies were applied onto cells and incubated at RT for 1 h. The following antibodies were used for immunofluorescence staining: mouse monoclonal FHOD1 (Santa Cruz sc-365437; 1:100), mouse Dia1 (BD biosciences 610848; 1:100), and rabbit monoclonal CAV-1 (CST 3267; 1:100). Alexa-conjugated phalloidin was added together with primary antibody solutions onto cells. All immunofluorescence data were obtained with upright Leica SP8 confocal microscope with HC PL APO 63×/1.30 GLYC CORR CS2 objective. The pixel size was optimized properly to achieve the maximum resolution, which was calculated to be 63.3 nm. The CAV-1 pit size was thus defined as no more than 2 × 2 pixel and the CAV-1-clustered rosette size was defined as no less than 3 × 3 pixel. For detection and measure of the cytoplasmic CAV-1-tagged vesicles, the ‘Spots’ tool of Imaris 9.2 (Bitplane) was used with the configuration defined as 2 μm for estimated XY diameter. The numbers and sizes of spots were calculated subsequently.

### Live cell imaging

Cells were plated prior to imaging on 10 μg/ml fibronectin-coated glass-bottomed dishes (MatTek Corporation). The time-lapse images of cells with transient transfection of CAV-1-mEGFP, mCherry-actin, and CAV-1-mCherry were acquired with 3I Marianas imaging system (3I intelligent Imaging Innovations), consisting of an inverted spinning disk confocal microscope Zeiss Axio Observer Z1 (Zeiss) and a Yokogawa CSU-X1 M1 confocal scanner. Appropriate filters, heated sample environment (+37°C), controlled CO_2_, and 63×/1.2 WC-Apochromat Corr WD = 0.28 M27 objective (Zeiss) were used. The images were acquired via SlideBook 7.0 software (3I intelligent Imaging Innovations) and recorded via Neo sCMOS (Andor) camera. The recording was set as every 1 sec for 10 min and one focal plane was recorded for all live cell videos. For tracking and speed measurement of CAV-1 vesicles, the Imaris 9.2 (Bitplane) ‘Track’ module with globular-objects over time was used as in previous study (Jiu, 2018). Then, 2 μm estimated XY diameter, 5 μm max distance, and 3 max gap size were set for analyzing.

### Western blotting

All cell lysates were prepared by washing the cells once with PBS and scraping them into lysis buffer (50 mM Tris–HCl, pH 7.5, 150 mM NaCl, 1 mM EDTA, 10% Glycerol, and 1% Triton X-100) supplemented with 1 mM PMSF, 10 mM DTT, 40 μg/ml DNase I, and 1 μg/ml of leupeptin, pepstatin, and aprotinin. All preparations were conducted at 4°C. Protein concentrations were determined with Bradford reagent (#500-0006, Bio-Rad Laboratories) and equal amounts of the total cell lysates were mixed with Laemmli Sample Buffer (LSB), boiled, and ran on 4%–20% gradient sodium dodecyl sulfate polyacrylamide gel electrophoresis gels (#4561096, Bio-Rad). Proteins were transferred to nitrocellulose membrane with Trans-Blot Turbo transfer system (Bio-Rad) using Mini TGX gel transfer protocol. Membrane was blocked in either 5% milk or 5% BSA in TBS with 0.1% Tween 20 (TBS-T) for 1 h at RT. Primary and secondary antibodies were diluted into fresh blocking buffer for incubation overnight at 4°C and 1 h at RT, respectively. Proteins were detected from the membranes with Western Lightning ECL Pro substrate (PerkinElmer). The following antibodies were used in this study: CAV-1 (D46G3) rabbit monoclonal antibody (1:1000 dilution; #3267, Cell Signaling), FHOD1 rabbit polyclonal antibody (1:1000 dilution; SAB4200147, Sigma-Aldrich), beta actin monoclonal antibody (1:5000 dilution; 66009-1-Ig, Proteintech), Dia1 rabbit polyclonal antibody (1:500 dilution; NBP2-14667, Novus Biologicals Inc.), and GAPDH mouse polyclonal antibody (1:1000 dilution; #G8795, Sigma-Aldrich).

### Plasmids, drugs, and hypo-osmotic shock

EGFP-C1, mCherry-actin, and actin-GFP were kind gifts from Pekka Lappalainen (University of Helsinki, Finland). CAV-1-mCherry (#27705) and CAV-1-mEGFP (#27704) were from Addgene. Active form of GFP-FHOD1 (1–1011) and GFP-Dia1 (ΔN3; 543–1192) were constructed by the overlap extension method, respectively. All plasmids were sequenced for verification. Drugs were dissolved in dimethyl sulfoxide as stock media and added directly to complete media (DMEM with 10% FBS) prior to experiments. SMIFH2 was used to inhibit actin filament elongation by inactivating formin activity (15 μM for 2 h). Hypo-osmotic medium was made by using complete growth medium diluted appropriately in deionized water (dilution 1:9 to obtain 30 mOsm) and used in all the experiments. Alternatively, dialyzed serum was added into diluted DMEM with deionized water, which was used to verify the phenotype.

### Real-time quantitative PCR

Total mRNA was extracted with GeneJET RNA purification kit (#K0731, Thermo Scientific) and single-stranded cDNA was synthetized (#K1671, Thermo Scientific) from 500 ng of extracted mRNA. The following primers were used: forward CAV-1 5′-AACCTCCTCACAGTTTTCATCC-3′, reverse CAV-1 5′-CTTGTTGTTGGGCTTGTAGATG-3′, forward GAPDH 5′-GAAGGTGAAGGTCGGAGTC-3′, and reverse GAPDH 5′-GAAGATGGTGATGGGATTTC-3′. Quantitative PCRs were carried out with Maxima SYBR Green/ROX (#K0221, Thermo Scientific) in Bio-Rad CFX96 (Bio-Rad). Changes in expression were calculated with 2^^ΔCt^ method and normalized to GAPDH and wild-type expression levels, respectively.

### Fabrication of soft substrate

Polyacrylamide (PAA) gels of various stiffness were prepared by mixing 40% PAA and 2% bis-acrylamide solution, as described previously (Jiu et al., 2019). Briefly, the gel solution for desired stiffness, ammonium persulfate, and tetramethylethylenediamine were mixed in 1000:10:1 proportion and then placed between a hydrophobic glass (octadecyltrichlorosilane-treated; 104817, Sigma-Aldrich) and the transparency sheet treated with 3-APTMS (A17714, Alfa Aesar). Once polymerized, the hydrophobic plate was carefully removed. The gel was conjugated with sulfosuccinimidyl 6 (4-azido-2-nitrophenyl-amino) hexanoate (sulfo-SANPAH) and incubated with rat tail type I collagen (25 µg/ml) (A1048301, Invitrogen) at 4°C for overnight.

### Statistical analysis

Statistical data analyses were performed with Excel (Microsoft) and Origin (Origin 2018b). Normality of the data was examined with the Shapiro–Wilk test and a quantile–quantile plot. For the data following normal distribution, Student’s two-sample unpaired *t*-test was used. If data did not follow normal distribution, Mann–Whitney *U*-test for two independent samples was conducted. For analyzing the proportion of CAV-1-tagged vesicle mobility type, 30 vesicles were randomly selected in each cell, and the trajectories and mean rates of the movements were calculated to define the percentage of ‘dwelling’ and ‘go and dwelling’ (Figure 1F). For analyzing the density of CAV-1, the total CAV-1 number of each cell was measured by Imaris ‘Spots’ tool, the area of each cell was measured by Fiji ImageJ, and the total number was divided by cell area to calculate the average number of CAV-1-positive vesicles per μm^2^ (Figures  2E and 3E). For analyzing the CAV-1 movement rate, the mean speed of each vesicle within 10 min live cell video was measured by Imaris 9.2 ‘Track’ module and pooled together to calculate the average rate with color bars indicating the tracked mean speed ranging from 0 to 0.3 μm/sec (Figures  2G, H and 3G, H). For analyzing the disappearance percentage of CAV-1-tagged vesicles upon hypo-osmotic shock, the total CAV-1 number of each cell was measured by Imaris 9.2 ‘Spots’ tool at 0, 2, and 5 min after hypo-osmotic shock and normalized to the number at 0 min.

## Supplementary material

Supplementary material is available at *Journal of Molecular Cell Biology* online.

## Supplementary Material

mjab070_Supplementary_Data
